# Distribution of Mobile Health Applications amongst Patients with Symptomatic Peripheral Arterial Disease in Germany: A Cross-Sectional Survey Study

**DOI:** 10.3390/jcm11030498

**Published:** 2022-01-19

**Authors:** Kastriot Alushi, Irene Hinterseher, Frederik Peters, Ulrich Rother, Moritz S. Bischoff, Spyridon Mylonas, Eberhard Grambow, Alexander Gombert, Albert Busch, Daphne Gray, Nikolaos Konstantinou, Konstantinos Stavroulakis, Marco Horn, Hartmut Görtz, Christian Uhl, Hannes Federrath, Hans-Heinrich Trute, Thea Kreutzburg, Christian-Alexander Behrendt

**Affiliations:** 1 Research Group GermanVasc, Department of Vascular Medicine, University Heart and Vascular Centre UKE Hamburg, University Medical Centre Hamburg-Eppendorf, 20246 Hamburg, Germany; k.alushi@uke.de (K.A.); f.peters@uke.de (F.P.); t.kreutzburg@uke.de (T.K.); 2 Berlin Institute of Health, Vascular Surgery Clinic, Charité-Universitätsmedizin Berlin, Freie Universität Berlin and Humboldt-Universität zu Berlin, 10117 Berlin, Germany; irene.hinterseher@mhb-fontane.de; 3 Brandenburg Medical School Theodor Fontane, 16816 Neuruppin, Germany; 4 Department of Vascular Surgery, University Medical Center Erlangen, 91054 Erlangen, Germany; ulrich.rother@uk-erlangen.de; 5 Department of Vascular and Endovascular Surgery, University Hospital Heidelberg, 69120 Heidelberg, Germany; moritz.bischoff@med.uni-heidelberg.de (M.S.B.); christian.uhl@med.uni-heidelberg.de (C.U.); 6 Department of Vascular and Endovascular Surgery, Medical Faculty, University of Cologne, 50937 Cologne, Germany; spyridon.mylonas@uk-koeln.de; 7 Department of General, Visceral, Thoracic, Vascular and Transplantation Surgery, Rostock University Medical Center, 18057 Rostock, Germany; eberhard.grambow@med.uni-rostock.de; 8 European Vascular Center Aachen Maastricht, Department of Vascular Surgery University Hospital RWTH Aachen, 52074 Aachen, Germany; agombert@ukaachen.de; 9 Division of Vascular and Endovascular Surgery, Department of Visceral, Thoracic and Vascular Surgery, Medical Faculty Carl Gustav Carus, University Hospital Carl Gustav Carus Dresden, Technische Universität Dresden, 01307 Dresden, Germany; albert.busch@uniklinikum-dresden.de; 10 Department of Vascular and Endovascular Surgery, Goethe University Hospital, 60590 Frankfurt, Germany; daphne.gray@kgu.de; 11 Department of Vascular Surgery, Ludwig Maximilians University Hospital, 80539 Munich, Germany; nikolaos.konstantinou@med.uni-muenchen.de (N.K.); konstantinos.stavroulakis@med.uni-muenchen.de (K.S.); 12 Division of Vascular and Endovascular Surgery, Department of Surgery, University Medical Center Schleswig-Holstein, 24105 Kiel, Germany; marco.horn@uksh.de; 13 Department of Vascular and Endovascular Surgery, Bonifatius Hospital Lingen, 49808 Lingen, Germany; hartmut@goertz-lingen.de; 14 Working Group Security in Distributed Systems at University of Hamburg, Department of Computer Science, University of Hamburg, 22527 Hamburg, Germany; federrath@informatik.uni-hamburg.de; 15 Faculty of Law, University of Hamburg, 20148 Hamburg, Germany; hans-heinrich.trute@uni-hamburg.de

**Keywords:** wearable electronic devices, smartphone, peripheral arterial disease

## Abstract

Background: Broadly available digital and mobile health applications (also known as mHealth) have recently gained increasing attention by the vascular community, but very little is known about the dissemination and acceptance of such technologies in certain target populations. The current study aimed to determine the user behaviour and acceptance of such digital technologies amongst patients with peripheral arterial disease (PAD). Methods: A cross-sectional survey of consecutively treated inpatients at 12 university institutions, as well as one non-university institution, was conducted. All admitted patients with symptomatic PAD were surveyed for 30 consecutive days within a flexible timeframe between 1 July and 30 September 2021. The factors associated with smartphone use were estimated via backward selection within a logistic regression model with clustered standard errors. Results: A total of 326 patients participated (response rate 96.3%), thereof 102 (34.0%) were treated for intermittent claudication (IC, 29.2% women, 70 years in median) and 198 were treated for chronic limb-threatening ischaemia (CLTI, 29.5% women, 70 years in median). Amongst all of the patients, 46.6% stated that they had not changed their lifestyle and health behaviour since the index diagnosis (four years in median), and 33.1% responded that they were not aware of the reasons for all of their medication orders. Amongst all those surveyed, 66.8% owned a smartphone (IC: 70.6%, CLTI: 64.1%), thereof 27.9% needed regular user support. While 42.5% used smartphone apps, only 15.0% used mobile health applications, and 19.0% owned wearables. One out of five patients agreed that such technologies could help to improve their healthy lifestyle. Only higher age was inversely associated with smartphone possession. Conclusions: The current survey showed that smartphones are prevalent amongst patients with peripheral arterial disease, but only a small proportion used mobile health applications and a considerable number of patients needed regular user support. Almost half of the patients did not change their lifestyle and one third were not aware of the reasons for their medication orders, emphasising room for improvement. These findings can further help to guide future projects using such applications to identify those target populations that are reachable with digital interventions.

## 1. Introduction

With more than 230 million patients, peripheral arterial disease (PAD) remains a major public health concern worldwide, owing to limb loss, mortality, and economic burden [1,2]. The data derived from large real-world registries suggest that long-term outcomes are devastating due to five-year amputation and death rates of 13–50% in patients with intermittent claudication (IC), and 50–90% with chronic limb-threatening ischaemia (CLTI) [3,4]. During a ten-year follow up after the index treatment for PAD, more than 7% of men and 4% of women were diagnosed with incident lung cancer [5]. Against this backdrop, it appears striking that even in high-volume quality improvement registries, the recent prevalence of active smoking as a common modifiable risk factor was reported by more than 44% of the patients [6]. Furthermore, even though all of the practice guidelines recommend an optimal pharmacological treatment on the grounds of high-level comparative effectiveness evidence [7,8,9], the prescription rates of statins remained considerably low in numerous international cohorts [10,11,12]. Other important pillars of best medical therapy, such as nutrition, were either not comprehensively covered by most valid guidelines or were prone to show low adherence rates in everyday practice [13]. This gap between the evidence-based guideline recommendations and the adherence to life- and limb-saving therapies for PAD patients may be bridged by new ways of patient-centred counselling and education. In 2013, a review of smartphone apps relating to major vascular disease documented the availability of 49 vascular-themed apps [14]. More recently, based on the novel concept of gamification, the Society for Vascular Surgery (SVS) piloted a mobile app for supervised exercise therapy as first line therapy for PAD patients [15]. The central question remains of how we can interact with the patient and subsequently improve the underlying health behaviour and modifiable-risk profile. Amongst others, supporting intrinsic motivators, as well as innovative concepts, such as “teachable moment” and “nudging behaviour”, may help with that important task [16,17]. Unfortunately, many previous efforts dissatisfied in terms of efficacy, cost effectiveness, and scalability [18,19,20]. Recently, the potential of broadly available digital health and mobile applications (also known as mHealth) have gained more attention by the cardiovascular community [21]. Those digital technologies, including mobile apps, wearables, and telehealth solutions, play an important role in healthcare delivery, patient-reported outcome research, and quality improvement in several cardiovascular diseases [22,23,24,25,26]. Due to the challenges related to the ongoing pandemic, the subsequent rise in the acceptance and adoption of digital technologies in all areas of life opened a unique window of opportunity for developing suitable health-based solutions in the coming years. However, the distribution of digital technologies amongst this considerably old and frail target population remains largely unknown beyond comparable age groups. In the current study, by using a survey approach, we aimed to determine the spread of smartphones and mobile health applications, as well as user behaviour in terms of healthcare education, amongst patients with PAD. Specifically, we aimed at quantifying the subgroup of PAD patients potentially amenable for digital interventions and at identifying factors associated with smartphone use.

## 2. Materials and Methods

This was a cross-sectional survey study of all suitable consecutively treated adult patients with inpatient treatment for either IC or CLTI, treated at the twelve participating university medical centres and one non-university institution in Germany. The study centres pledged to include all incomers without selection by the treating physician. Patients with severe dementia and patients who were not able to understand the questionnaire due to language barriers were excluded. Drawing on the experience from earlier survey-based registry studies and a preliminary acceptance test at two university institutions (Hamburg, Heidelberg), a pragmatic one-page questionnaire was developed so that the target group could be reached with a high response rate (Supplementary Table S1). The utilised questionnaire consisted of 15 items in 3 sections. Variables included gender identity (men, women, diverse), age in years, time in years since first PAD diagnosis, disease stage (CLTI vs. IC), highest educational degree (prefer not to say, no associate degree, final apprenticeship examination, technical college degree, diploma, bachelor’s degree or similar, master’s degree or similar, promotion/thesis), living area (rural, urban, intermediate), preferred sources of health education (television, newspaper, internet, radio, other), the patient changed health behaviour since first PAD diagnosis (yes, no), the patient owns a smartphone (yes, no), the patient uses mobile applications (“apps”) (yes, no), the patient uses mobile health applications (yes, no), the patient needs regular user support to use the smartphone (yes, no), the patient owns a wearable (yes, no), patients’ agreement that mobile health applications and other digital technologies that observe and process health behaviour may help them to live healthier (yes, no, maybe), the patient knows the reason for all medication orders (yes, no, maybe) (Supplementary Table S1).

For minimising interviewer bias, a German language study protocol was written a priori and circulated with the study invitation amongst all centres (available from the authors upon request). We applied clustered consecutive sampling in the 13 vascular surgery facilities in Germany to account for regional disparities.

German study centres in Aachen, Berlin, Dresden, Erlangen, Frankfurt, Hamburg, Heidelberg, Cologne, Lübeck, Munich, Rostock, Lingen, and Neuruppin were invited to survey all patients within a timeframe of 30 days with flexible start and end dates between 1 July and 30 September 2021 (3 months). The paper-based survey was conducted either by a trained study nurse or the local site principal investigator during the admission day, before the index treatment. We adhered to the Consensus-Based Checklist for Reporting of Survey Studies (CROSS) for reporting the study results [27].

### 2.1. Ethical Considerations

Due to the anonymized nature of the deidentified study data, no ethical approval was necessary.

### 2.2. Statistical Analysis

The survey data was collected on a paper base and subsequently digitalised by the first author in the coordinating centre (Hamburg, Germany). We summarized the baseline characteristics of the patients with medians and interquartile ranges (IQR) for age and with percentages and a 95% confidence interval (CI) for categorical variables. Missing data (ranging between 16% for time since PAD diagnosis and 0% for highest education level, Table 1) were handled by pairwise exclusion (Table 1). For assessing the selectivity of our sample, we compared the study characteristics with data collected in a tertiary non-university centre (Lingen, Germany) and an outpatient facility (Hamburg, Germany) using the same selection criteria (Supplementary Table S2). Odds ratios (OR) are presented with a 95% CI. To address the impact of certain variables on the odds of using a smartphone, multivariable logistic regression models were developed, including the following variables in a stepwise backward elimination (Wald) of variables: higher age (in years), female sex (vs. male sex), no educational degree, urban living area (vs. rural or intermediate), chronic limb-threatening ischaemia (vs. intermittent claudication). To account for clustering in the data (by centre), we applied the complex samples regression module in SPSS (IBM Corporation, New York, NY, USA). Data processing was performed with SPSS version 25.

### 2.3. Data Sharing Statement

The data that support the findings of this study are available from the corresponding author, C.-A.B., upon reasonable request.

## 3. Results

Between 1 July and 30 September 2021, a total of 13 participating study centres submitted 326 completed surveys from individual patients, thereof 102 (34.0%) were treated for IC (29.2% women, 70 years in the median, IQR 62–78) and 198 were treated for CLTI (29.5% women, 70 years in the median, IQR 62-79) (Table 1).

The baseline characteristics of the entire cohort and by smartphone use are presented in Table 1. Less than 5% (*n* = 12) of the eligible patients declined participation, resulting in a response rate of 96.3%. In terms of the highest education level, 53.7% had finished their apprenticeship, while 11.3% stated that they had no educational achievement. Most of the patients came from an urban (52.3%) or rural living area (33.7%).

When being asked about the preferred source of health-related information (multi-choice), most of the patients selected television (12.3%), followed by internet (8.0%), and newspapers (4.3%). A total of 146 patients (46.6%) stated that they had not changed their lifestyle and health behaviour since they were first diagnosed with PAD (four years in median, IQR 1.5-10). A total of 105 patients (33.1%) responded that they were not aware of the specific reasons for all of their medication orders.

### 3.1. Overall Smartphone Use

Amongst all of the surveyed patients, 66.8% owned a smartphone and thereof 27.9% needed regular user or application support to use the smartphone.

### 3.2. Differences by Clinical Disease Stage

As compared with CLTI, the patients treated for IC more frequently owned a smartphone (70.6% vs. 64.8%), less often needed regular user support to use the smartphone (23.8% vs. 29.0%), more often used mobile applications (52.5% vs. 39.1%), and mobile health applications (15.7% vs. 15.1%), as well as wearables (28.4% vs. 13.9%). While 27.7% of the patients with IC estimated that digital or mobile health applications could help to improve their modifiable-risk profile and health behaviour, the approval was only 18.1% amongst the patients with CLTI.

### 3.3. Differences by Educational Status

The proportion of patients who owned a smartphone was the lowest in the group without educational achievement (59.5%), followed by apprenticeship (64.0%). The lowest prevalence of mobile health application use was observed in the patients without educational achievement (19.0%), followed by the patients who finished an apprenticeship (15.3%) (results not shown in tables).

### 3.4. Factors Associated with Smartphone Use

In the multivariate analysis, only higher age was associated with lower odds of owning a smartphone. There was no significant difference in smartphone prevalence observed between men and women (Table 2).

### 3.5. Sensitivity Analyses and Generalisability

In the sensitivity analyses, the confidence intervals of the baseline characteristics between the university institutions vs. the non-academic medical centre vs. the outpatient facility were overlapping (Supplementary Table S2).

## 4. Discussion

In this survey of 326 patients who were consecutively treated at the 13 vascular centres in Germany, approximately two out of three owned a smartphone. The smartphone users were younger and were more often women, better educated, more often lived in urban living areas, and suffered from IC.

Interestingly, almost one third of the smartphone owners needed regular user or application support, and only a small proportion of the patients used mobile health applications, while television, followed by the internet, were the most used sources to gather health-related information. Less than 30% of the patients with IC, and 20% with CLTI, believed that such applications may help them to ameliorate their modifiable-risk profile and health behaviour. Strikingly, one out of three patients in this high-volume quality improvement collaboration responded that they did not know the reasons for all of their medication orders, emphasising a missed opportunity to improve drug adherence.

These findings, along with a commonly known healthy user bias in previous clinical trials on PAD, emphasise that the utilisation of mobile health applications and other digital technologies in both clinical care and research may miss an underprivileged subpopulation.

The utilisation of wearables and mobile health technologies in clinical trials on supervised exercise has previously demonstrated satisfying results in patients with PAD [28]. In addition, an increasing number of mobile health applications for commonly used smartphone platforms has been introduced to the market [25], but their area of the application remains limited to basic education and follow-up [29,30], wound documentation or telecare for surgical site infection [31], as well as activity tracking [32]. To date, there is neither a broad consensus on the scope of the educational content nor how it should be presented to the target audience.

As of 2020, only 5% of the European Union inhabitants between 16 and 74 years used so-called smart health devices that monitor blood pressure, blood glucose, or body weight. Approximately 19% used wearables, such as smartwatches or fitness bracelets. Meanwhile, the proportion of German inhabitants aged 70 years or above using smartphones was 52.1% (source: Eurostat Online Database, accessed on 1 October 2021). Against this backdrop, the results of the current survey appear surprising, since 71% of patients with IC, and 65% with CLTI declared that they owned a smartphone, which is a slightly higher proportion than in the age-matched populations. While the prevalence of wearables was higher in the patients with IC (28%), fewer patients owned wearables in the CLTI group (13%).

The utilisation of mobile health applications in vascular care may offer interesting opportunities to interact with a markedly vulnerable target population, but we must consider that, those who are under high risk (e.g., the elderly males with low educational level), may not be reached by digital technologies. Thus, providers should adapt a patient-based approach to such digital technologies, as with other more traditional interventions and treatments, where risk scores were used to identify suitable target groups. It should be also emphasised that mobile health applications could complement the physician–patient interaction, which was frequently accused of being insufficient, especially beyond discharge from the hospitals. Most recently, the Society for Vascular Surgery (SVS) launched the SVS interactive practice guideline app, which primarily addressed vascular physicians but also vascular patients. This common approach to combine content for experts, as well as patients and their relatives, may improve a broader spread of mobile health applications in the care of vascular disease. Our findings may be a motivation for healthcare providers in Germany and beyond to strengthen their efforts in implementing such technologies to be used by the elderly. Furthermore, the survey results emphasised that there may be a great chance to reach those patients who had not changed their lifestyle and were not sufficiently informed about the importance of the best medical treatment to improve their long-term outcomes. By improving the awareness and providing access to the appropriate digital applications with evidence-based lifestyle advice, the generally low acceptance may change over time.

In a next step, we will use the current study findings to design a prospective interventional study that aims to determine the potential benefits of such digital technologies to improve the adherence to best medical treatment and the corresponding outcomes in the longer term.

The current study has several strengths but also limitations. Firstly, our study was designed as a survey study to gather complete responses from a target population known to decline comprehensive questionnaires. In the prospective GermanVasc cohort study (NCT03098290), only 73% of the patients who were enrolled during the index treatment completed the questionnaire on patient-reported outcomes, and only 21% accepted to complete the questionnaire at the 12 months follow-up. One of the most frequently documented reasons was that the questionnaires were too complicated [6]. Hence, we pre-tested different versions of the paper-based survey in two institutions and decided to use a one-page questionnaire to reach the highest response rate. Secondly, while numerous centres of different care levels are involved in the treatment of this target population, only university institutions were invited to participate in the current study for practical reasons. In order to assure a complete and valid survey amongst all inpatients, certain infrastructural specifications are necessary, which makes it more likely that university institutions match all assumptions. Consequently, our results are likely more generalizable for the typical patient population in high-volume centres, e.g., a higher share of men and CLTI patients. In order to quantify the possible selection bias, we compared the results with a non-university centre and another outpatient population, overall, not suggesting any fundamental differences amongst the patient characteristics. Lastly, there are likely numerous confounders and comorbidities associated with smartphone use that have not been addressed appropriately in the current survey study. In a future study, the relevant obstacles for smartphone use in the subgroups should be addressed in order to increase the dissemination of digital technologies used in health care.

## 5. Conclusions

The current survey showed that smartphones are prevalent amongst patients with peripheral arterial disease, but only a small proportion used mobile health applications and a considerable number of the patients needed regular user support. Almost half of the patients had not changed their lifestyle and one third were not aware of the reasons for their pharmaceutical prescriptions, emphasising room for improvement. These findings can further help to guide future projects using such technologies to identify those target populations that are reachable with digital interventions.

## Figures and Tables

**Table 1:** Baseline characteristics of the entire cohort (*n* = 326 patients) and stratified by smartphone use. PAD: Peripheral Arterial Disease. N/A: Not applicable/available.

	Total Cohort	Smartphone	Smartphone
		Yes	No
Number of patients, *n*/N (%)	326/326 (100.0)	215/322 (66.8)	107/322 (33.2)
Female sex, *n*/N (%)	92/314 (29.3)	63/211 (29.9)	27/99 (27.3)
Median age, years (interquartile range), *n*	70 (62–78), 322	67 (60–74), 212	78 (70–84), 106
Chronic limb-threatening ischaemia, *n*/N (%)	198/300 (66.0)	127/199 (63.8)	69/99 (69.7)
Does not know the reason for all medication orders, *n*/N (%)	105/317 (33.1)	53/211 (25.1)	52/105 (49.5)
Median time since first PAD diagnosis, years (interquartile range), *n*	4 (1.5–10) *n* = 274	4 (1.5–8) *n* = 186	3.75 (1–10) *n* = 88
Lifestyle not changed since first PAD diagnosis, *n*/N (%)	146/313 (46.6)	77/208 (37.0)	69/104 (66.3)
Highest education level, *n*/N (%)			
Prefer not to say	38/326 (11.7)	19/215 (8.8)	15/107 (14.0)
No associate degree	37/326 (11.3)	22/215 (10.2)	15/107 (14.0)
Final apprenticeship examination	175/326 (53.7)	112/215 (52.1)	63/107 (58.9)
Technical college degree	31/326 (9.5)	26/215 (12.1)	5/107 (4.7)
Diploma	28/326 (8.6)	21/215 (9.8)	7/107 (6.5)
Bachelor’s degree or similar	4/326 (1.2)	3/215 (1.4)	1/107 (0.9)
Master’s degree or similar	11/326 (3.4)	10/215 (4.7)	1/107 (0.9)
Promotion	2/326 (0.6)	2/215 (0.9)	0/107 (0)
Living region, *n*/N (%)			
Rural	101/300 (33.7)	65/215 (32.3)	36/107 (36.7)
Intermediate	42/300 (14.0)	29/215 (14.4)	13/107 (13.3)
Urban	156/300 (52.3)	107/215 (53.2)	49/107 (50.0)
Preferred source of health-related information (multi-choice), *n*/N (%)			
Television	40/326 (12.3)	20/215 (9.3)	19/107 (17.8)
Internet	26/326 (8.0)	24/215 (11.2)	2/107 (1.9)
Radio	5/326 (1.5)	2/215 (0.9)	3/107 (2.8)
Newspaper	14/326 (4.3)	5/215 (2.3)	9/107 (8.4)
Other	27/326 (8.3)	18/215 (8.4)	9/107 (8.4)
Mobile application use, *n*/N (%)	135/318 (42.5)	134/213 (62.9)	N/A
Mobile health application use, *n*/N (%)	48/319 (15.0)	47/213 (22.1)	N/A
Needs regular user support to use smartphone, *n*/N (%)	86/319 (27.0)	60/215 (27.9)	N/A
Wearables, *n*/N (%)	61/321 (19.0)	57/213 (26.8)	4/107 (3.7)
Agreement that mobile health applications can affect lifestyle positively, *n*/N (%)			
Yes	65/316 (20.6)	56/209 (26.8)	8/106 (7.5)
No	154/316 (48.7)	79/209 (37.8)	75/106 (70.8)
Maybe	97/316 (30.7)	74/209 (35.4)	23/106 (21.7)

**Table 2:** Variables associated with smartphone use in *n* = 267 (of 326) patients included in this cross-sectional survey study. The model was weighted to account for clustering by centres and adjusted for higher age, female sex, chronic limb-threatening ischaemia, urban living area, and no educational degree.

	Odds Ratio	95% Confidence Interval	*p*-Value
Higher age (increase by one year)	0.89	0.86–0.92	<0.001
Female sex (vs. male sex)	1.81	0.92–3.56	0.081
No educational degree	0.70	0.24–2.05	0.480
Urban living area	0.83	0.45–1.53	0.522
Chronic limb-threatening ischaemia (vs. intermittent claudication)	0.86	0.40–1.82	0.659

## Data Availability

The data that support the findings of this study are available from the corresponding author, C.-A.B., upon reasonable request.

## Supplementary Materials

The following supporting information can be downloaded at: https://www.mdpi.com/article/10.3390/jcm11030498/s1. Supplementary Table S1: Survey items and answering options, Supplementary Table S2: Comparison of baseline characteristics between 12 university institutions, one non-academic institution, and an outpatient cohort. CI: Confidence interval. OPT: Optimal pharmacological treatment.
