# Supplementary material for: Distribution of Mobile Health Applications amongst Patients with Symptomatic Peripheral Arterial Disease in Germany: A Cross-Sectional Survey Study
Source: J Clin Med. 2022 Jan 19;11(3):498. doi: 10.3390/jcm11030498 (PMC8836389; doi:10.3390/jcm11030498)
Supplement: Supplementary file 1 [file jcm-11-00498-s001.zip › jcm-1526312-supplementary.pdf]

Electronic Supplement

Table S1: Survey items and answering options

1	**Gender identity**	Men, women, diverse
2	**Age**	In years
3	**Peripheral arterial disease diagnosed (known) since**	In years
4	**Chronic limb-threatening ischaemia (wound healing disorders, diabetic foot ulcers)**	Yes, no
5	**What is your highest education degree?**	Prefer not to say, no associate degree, final apprenticeship examination, technical college degree diploma, bachelor's degree or similar, master's degree or similar, promotion (thesis)
6	**What is your main living region?**	Rural, intermediate, urban
7	**What is your preferred source of health-related information?**	Television, internet, newspaper, radio, other
8	**Have you changed your health behaviour since you were informed about the diagnosis peripheral arterial disease?**	Yes, no
9	**Do you own a smartphone?**	Yes, no
10	**If yes: Do you use mobile applications ("apps")?**	Yes, no
11	**Have you ever used health-related digital and mobile applications ("mHealth")?**	Yes, no
12	**Do you need regular user support (assistance) to use your smartphone?**	Yes, no
13	**Do you own a wearable (e.g., smartwatch, heart rate monitor, pedometer)?**	Yes, no
14	**What is your opinion: Could mHealth and technologies which observe and process your behaviour help to improve your healthy lifestyle?**	Yes, maybe, no
15	**Do you know the reason for each of your medical prescriptions?**	Yes, maybe, no

Table S2: Comparison of baseline characteristics between 12 university institutions, one non-academic institution, and an outpatient cohort. CI: Confidence interval. OPT: Optimal pharmacological treatment.

	**GermanVasc Kotov et al. 2021**	**BARMER Claims Peters et al. 2020**	**University institutions**	**Non-academic institution**	**Outpatient facility**
**Number of patients**	5608	83867	298	28	47
**Female sex, % (95% CI)**	34.0%	45.8%	28.9 (23.7-34.5)	33.3 (16.5-54.0)	42.6 (28.3-57.8)
**Median age, years (interquartile range)**	69	71.9	70 (62-78)	69.5 (64.5-79.75)	74 (68-80)
**Chronic limb-threatening ischaemia, % (95% CI)**	29.9%	46%	65.3 (59.4-71.0)	73.1 (52.2-88.4)	45.7 (30.9-61.0)
**Smartphone use, % (95% CI)**	NA	NA	67.0 (61.3-72.4)	64.3 (44.1-81.4)	57.4 (42.2-71.7)
